# Association between the severity of gingival inflammation and microbial findings in children

**DOI:** 10.3389/fdmed.2025.1638435

**Published:** 2025-07-03

**Authors:** Hristina Tankova

**Affiliations:** Department of Pediatric Dentistry, Medical University Sofia, Sofia, Bulgaria

**Keywords:** gingivitis, microbial load, children's gingival health, periodontopathogens, subgingival microbiome

## Abstract

**Introduction:**

The oral cavity is home to hundreds of distinct microbial species, and specific periodontal pathogens are isolated from different ecological niches. Present study aimed to investigate the relationship between the severity of gingival inflammation and the presence of subgingival microorganisms in children with dental biofilm induced gingivitis.

**Material and Methods:**

The study included 30 children aged 12–14 years, divided into two groups based on the extent of gingival inflammation: **Group I**—16 children with BOP up to 30%; **Group II**—14 children with BOP over 30%. All children were interviewed to assess oral hygiene habits. Clinical examination was performed using an electronic periodontal probe, and the following were recorded: oral hygiene status (FMPS) and gingival status through BOP and SBI. For quantitative assessment of subgingival periodontopathogens, a genetic method - PCR—Real Time was used, and the following microorganisms were examined: *Aggregatibacter actinomycetemcomitans*, *Porphyromonas gingivalis*, *Treponema denticola*, *Tannerella forsythia*, *Prevotella intermedia*, *Peptostreptococcus micros*, *Fusobacterim nucleatum*, *Eubacterium nodatum*, *Capnocytophaga gingivalis*. The critical significance level for testing the null hypothesis was set at *α* = 0.05, corresponding to a 95% confidence level.

**Results:**

The majority of children showed improper oral hygiene habits. Children with generalized gingival inflammation had significantly higher plaque accumulation index values compared to those with localized inflammation. In children with generalized gingival inflammation, the quantities of all isolated periodontopathogens were higher compared to those with localized inflammation, which was also confirmed regarding the overall microbial load. *A. actinomycetemcomitans* was not isolated in children with localized gingival inflammation, while *T. denticola* was isolated in significantly lower quantities compared to generalized inflammation. *P. intermedia* and *P. micros* were isolated in significantly higher quantities in more severe gingival inflammation. In children with localized gingival inflammation, combinations of an average of 2 microorganisms were found in microbial complexes, while in children with generalized inflammation, microorganisms were twice as many and in more complex combinations.

**Conclusion:**

The microbial diversity within the subgingival biofilm significantly increases with disease severity, providing further evidence for the critical role of microbial ecology in the pathogenesis of gingival inflammation in children.

## 1 Introduction

The oral cavity harbors the second most diverse microbial ecosystem in the human body after the gastrointestinal tract, accommodating over 700 different bacterial species (1). These microorganisms colonize various niches within the oral environment, including the teeth, tongue, periodontal structures, and buccal mucosa, all interconnected through saliva (2). Interactions between the oral microbiome and the human host during early stages of development play a crucial role in shaping innate and adaptive immune functions, as well as physiological maturation, which in turn influence long-term health outcomes (3).

Dental biofilm is defined as a highly organized microbial community composed of cells attached to the tooth surface or to each other, embedded within an extracellular matrix (4). The dominant early colonizers of dental biofilm are oral streptococci, primarily from the *Streptococcus mitis* group, followed by gram-positive rods from the genus *Actinomyces*. Over time, gram-negative cocci and rods integrate into the initially gram-positive biofilm. The microorganisms forming the biofilm gradually adapt their phenotype in response to environmental changes within the community, gaining enhanced capacity for rapid growth, adaptation, and increased pathogenic potential (5, 6).

The oral cavity is home to hundreds of distinct microbial species, and specific periodontal pathogens are isolated from different ecological niches (7). In the context of periodontal pathology, the composition of microbial species in both supra- and subgingival biofilms undergoes significant shifts (8, 9).

Traditionally, the identification of individual microbial species and the study of their interactions with the host have relied on various culture-based methods (10). These techniques involve cultivating microorganisms under laboratory conditions, allowing for detailed analysis of their biochemical and physiological properties, such as metabolic functions and antibiotic resistance. Although foundational to microbiology, these methods have substantial limitations—particularly in detecting anaerobic bacterial species that are difficult to culture under standard laboratory conditions. Studies have shown that traditional culturing techniques may fail to detect a significant portion of the oral microbiome, with estimates suggesting that оnly around half of oral bacteria can be grown in the laboratory using conventional culture methods (11). There are significant difficulties in using cultural methods to characterize the periodontal microbiota. Anaerobic bacteria, which predominate in the subgingival space, are slow-growing and require a specific nutritional environment for growth and development. The identification of isolates is a sensitive method that requires the expertise of experienced microbiologists. All these limitations call for the search for alternative diagnostic methods for periodontopathogens through genetic techniques helps overcome this limitation by allowing scientists to detect bacteria that cannot be cultured, leading to a more complete understanding of the oral microbiome (11).

Modern genetic identification techniques such as **Polymerase Chain Reaction (PCR)**, regarded as the most effective method for detecting periodontal pathogens without the need for culturing, can identify a wide range of species even when present in minimal quantities (12).

A review of the global literature reveals that most studies focus on oral microorganisms in adult patients with inflammatory-destructive periodontal changes. In contrast, studies involving children remain relatively limited and are predominantly centered around dental caries (13–15). This provides a strong rationale for investigating the current state of the subgingival biofilm in children, specifically in the context of their periodontal health. **Aim:** The aim of the present study is to investigate the relationship between the severity of gingival inflammation and the presence of subgingival microorganisms in children aged 12–14 years with dental biofilm induced gingivitis.

## 2 Materials and methods

### 2.1 Participants

The study was conducted with a sample of 30 children aged 12–14 years from the city of Sofia. Before conducting the study, a power analysis was performed to determine the appropriate sample size. We selected a power of 0.80, with a significance level of 0.05 and an expected medium effect size (Cohen's d = 0.5). The power analysis indicated that a sample size of 30 children would be sufficient to detect statistically significant effects if they exist. This approach ensures that the study is adequately powered to identify real differences while minimizing the likelihood of Type II errors.

The inclusion criteria for participation were as follows:

• Signed informed consent for participation in the study, provided by a parent or legal guardian, and approved by the Ethics Committee of the Medical University (KENIMUS), protocol № 12/14.05.2020;
• Presence of provoked gingival bleeding involving more than 10% of gingival units, which, according to the current classification of periodontal diseases, categorizes the patient as having dental biofilm induced gingivitis (16);
• Absence of systemic diseases that pose a risk to periodontal health;
• No ongoing orthodontic treatment at the time of the study;
• No antibiotic use within the last three months.

The participants were grouped based on their severity of provoked gingival bleeding scores. A stratified random sampling method was used to select participants from the target population to ensure a representative sample.After the selection process, and for the purpose of the study, the children were categorized into two groups based on the current classification of gingival diseases and the extent of provoked gingival bleeding:

• **Group I:** 16 children with bleeding on probing (BOP) up to 30%—*localized gingival inflammation* (16);
• **Group II:** 14 children with BOP over 30%—*generalized gingival inflammation* (16).

## 3 Methodology

### 3.1 Clinical method

Clinical examinations were conducted in a dental office setting. Initially, anamnesis was collected to identify behavioral risk factors related to the children's oral hygiene habits.

Oral hygiene status was assessed using the Full Mouth Plaque Score (FMPS) index, through staining the teeth with a plaque-disclosing solution. The presence or absence of dental biofilm was recorded for all fully erupted permanent teeth at four gingival sites—distobuccal, mesiobuccal, buccal, and oral. The FMPS was calculated automatically by the software of the electronic periodontal probe and reflected the relative percentage of tooth surfaces covered by dental biofilm.

The clinical periodontal examination was performed using an electronic periodontal probe (PA ON, Orange dental GmbH & Co. KG Germany) by one examiner (H.T.). To ensure reliability in probing and index scoring the examiner participated in a training session where reviewed detailed scoring criteria and probing techniques with electronic periodontal probe. Also, the electronic periodontal probe used in this study has an automatic calibration system that ensures consistent measurements across all trials, eliminating the potential for examiner-related inconsistencies.

The indices used in the study were as follows:

• **Bleeding on Probing (BOP).** All fully erupted permanent teeth were probed at four sites (mesiobuccal, buccal, distobuccal, and oral). The presence or absence of bleeding was recorded, and the index represented the relative percentage of gingival units with provoked bleeding.
• **Sulcus Bleeding Index (SBI).** Probing was performed orally in quadrants one and three, and bucally in quadrants two and four. The index was recorded on a scale from 0 to 5, yielding a mean score reflecting the severity of gingival inflammation (17).

Data entry from probing was manually conducted by the operator using the control buttons on the probe, while the calculation of indices was automatically processed by the software (Softwear byzz, version 6.2.5, licensed for FDM Sofia).

### 3.2 Genetic method

To assess microbial load and provide quantitative characterization of subgingival periodontal pathogens, a genetic method—Real-Time Polymerase Chain Reaction (PCR) (MIP Pharma GmbH, Germany)—was employed.

PCR is a method used to amplify DNA *in vitro*. Real-time PCR incorporates fluorescence-based detection, using intercalating dyes (e.g., SYBR Green) or fluorescently labeled probes (e.g., TaqMan probes). This allows real-time monitoring of DNA amplification, with fluorescence intensity proportional to the amount of DNA replicated. The amplification mechanism is identical to conventional PCR, with final quantification computed via dedicated software. Microbial quantification was based on Ct values. The PCR reaction was performed in real-time, and the Ct values for the target genes were recorded. These Ct values were then used to estimate microbial abundance by comparing them to a pre-determined standard according to the company performing PCR.

For the purposes of this study, nine reference strains of subgingival microorganisms were analyzed, grouped into four microbial complexes according to Socransky's classification (18) (as defined by the manufacturer, MIP Pharma GmbH, Germany):

• **Purple Complex:** Aggregatibacter actinomycetemcomitans
• **Red Complex:** Porphyromonas gingivalis, Treponema denticola, Tannerella forsythia
• **Orange Complex:** Prevotella intermedia, Parvimonas micra (formerly P. micros), Fusobacterium nucleatum, Eubacterium nodatum
• **Green Complex:** Capnocytophaga gingivalis

For each child, up to five teeth exhibiting the most severe pathology were selected. The collection method involved inserting a sterile paper point into the chosen gingival sulcus, waiting for 5 s, then removing the paper point and placing it in a sterile test tube. All 5 paper points were collected together to form a pooled sample. The quantity of isolated pathogens and the composition of microbial associations were subsequently assessed. According to the company quantifying microbial load was done through quantitative PCR (qPCR) method.

### 3.3 Statistical methods

Data analysis was performed using **IBM SPSS (Version 19.0)** and **Microsoft Excel 2019**. The critical level of statistical significance for testing the null hypothesis (H₀) was set at ***α*** **=** **0.05**.

The following statistical methods were applied to ensure the objectivity of the analytical results:

• Descriptive analysis
• Analysis of variance (ANOVA)
• Independent samples t-test
• Pearson Chi-square test (*χ*^2^).

## 4 Results

### 4.1 Clinical findings

#### 4.1.1 Oral hygiene habits and oral hygiene Status

Among the behavioral risk factors, the oral hygiene habits of the participating children were evaluated. The following table presents data on the frequency and duration of oral hygiene procedures among the children in the two study groups (Table 1).

**Table 1:** Oral hygiene habits of children in the Two study groups.

OH habits BOP	Brushing frequency		Duration of oral hygiene procedure		
	Regular	Irregular	1 min	2 min	Time not tracked
BOP up to 30%	25%	75%	38%	6%	56%
BOP over 30%	36%	64%	57%	14%	29%
Общо	30%	70%	47%	10%	43%
	*Pearson χ^2^* *=* *2,314 p* *>* *0,05*		*Pearson χ^2^* *=* *2,420 p* *>* *0,05*		

From the table, it is evident that one-third of the children brush their teeth regularly (morning and evening), while 70% perform oral hygiene procedures only occasionally. Only 10% of all children brush their teeth for the recommended duration of two minutes, whereas 90% either do not track the time at all or brush for no more than one minute.

Based on the results, it can be concluded that, regardless of the severity of gingival inflammation, the majority of children exhibit inadequately established oral hygiene habits (*p* > 0.05).

The presence and quantity of supragingival biofilm serve as a prerequisite for the formation of subgingival biofilm, which is a leading etiological factor in the pathogenesis of plaque-induced gingivitis. The next table presents the oral hygiene status of the children in both study groups (Table 2).

**Table 2:** Oral hygiene Status of children in the Two study groups.

OH status BOP	N child	FMPS %
		Mean ± SD
BOP up to 30%	16	59,50 ± 28,08
BOP over 30%	14	67,93 ± 16,06
Total	30	62,26 ± 22,80
*Independent T test*	*t* *=* *9,896 р* *<* *0,05*	

From the table, it is evident that plaque accumulation among all examined children averages 62% of the tooth surfaces. Notably, children with generalized gingival inflammation exhibited significantly higher plaque accumulation—68%, compared to 60% in children with localized inflammation (*p* < 0.05).

#### 4.1.2 Gingival status and microbial load

The periodontal examination included an assessment of the extent of provoked gingival bleeding (BOP) and the severity of gingival inflammation (SBI) using the indices integrated into the electronic periodontal probe. The following table presents the data on gingival inflammation severity and total microbial load in children from both study groups (Table 3).

**Table 3:** Severity of gingival inflammation and microbial load.

Gingival status BOP	N child	SBI Mean ± SD	Microbial load Mean ± SD
BOP up to 30%	16	1,02 ± 0,73	1,5 × 10^8^ ± 1,4 × 10^8^
BOP over 30%	14	1,37 ± 0,81	9,6 × 10^8^ ± 1,7 × 10^9^
*Independent T test*		*t* *=* *0,434 р* *>* *0,05*	*t* *=* *10,552 р* *<* *0,05*

From the table, it can be observed that as the relative proportion of provoked gingival bleeding increases, there is a corresponding increase in the severity of gingival inflammation, although this relationship did not reach statistical significance (*p* > 0.05). It is also noteworthy that the total microbial load in children with localized gingival inflammation is significantly lower compared to that in children with generalized inflammation (*p* < 0.05).

### 4.2 Microbiological findings

#### 4.2.1 Quantitative characteristics of isolated periodontal pathogens

The following table presents the quantitative characteristics of the isolated periodontal pathogens, categorized by the study groups (Table 4).

**Table 4:** Quantities of isolated periodontal pathogens.

	BOP up to 30%	BOP over 30%	*ANOVA*
*A. actinomycetmcomitans*	–	1,5 × 10^3^ ± 1,5 × 10^3^	
*P. gingivalis*	2,4 × 10^5^ ± 1,5 × 10^5^	2,5 × 10^5^ ± 9,4 × 10^4^	*t* *=* *−1,37 p* *>* *0,05*
*T. denticola*	4,8 × 10^4^ ± 3,8 × 10^4^	1,7 × 10^5^ ± 2,4 × 10^5^	*t* *=* *4,66 p* *<* 0,05*
*T. forsythia*	2,1 × 10^3^ ± 1,6 × 10^3^	6,1 × 10^3^ ± 3,1 × 10^3^	*t* *=* *1,48 p* *>* *0,05*
*P. intermedia*	8,4 × 10^4^ ± 7,3 × 10^4^	3,0 × 10^5^ ± 4,5 × 10^5^	*t* *=* *4,28 p* *<* 0,05*
*P. micros*	6,3 × 10^2^ ± 5,8 × 10^2^	2,7 × 10^3^ ± 2,7 × 10^3^	*t* *=* *2,74 p* *<* 0,05*
*F. nucleatum*	1,1 × 10^3^ ± 1,1 × 10^3^	1,2 × 10^3^ ± 7,0 × 10^2^	*t* *=* *0,35 p* *>* *0,05*
*E. nodatum*	3,4 × 10^2^ ± 3,2 × 10^2^	7,2 × 10^2^ ± 5,4 × 10^2^	*t* *=* *1,46 p* *>* *0,05*
*C. gingivalis*	4,2 × 10^3^ ± 3,6 × 10^3^	3,1 × 10^4^ ± 5,2 × 10^4^	*t* *=* *2,98 p* *<* 0,05*

* Indicates the presence of a statistically significant difference.

The results from the table show that in children with generalized gingival inflammation, the quantities of all isolated periodontal pathogens are higher compared to those with localized inflammation, which was also confirmed in terms of overall microbial load.

It is noteworthy that among all tested periodontal pathogens, *A*. *actinomycetemcomitans* was the only species not isolated in children with localized gingival inflammation.

Among the red complex species described by Socransky, *T. denticola* was isolated in significantly lower quantities in children with localized inflammation (4.8 × 10^4^ ± 3.8 × 10^4^) (*p* < 0.05). In contrast, *P. gingivalis* showed similar levels regardless of the extent of gingival inflammation. *T. forsythia* was also isolated in higher quantities in cases of more severe inflammation, although this difference did not reach statistical significance (*p* > 0.05).

Within the orange complex, *P. intermedia* (3.0 × 10^5^ ± 4.5 × 10^5^) and *P. micros* (2.7 × 10^3^ ± 2.7 × 10^3^) were found in significantly higher quantities in cases of more severe gingival inflammation (*p* < 0.05). In contrast, differences in the quantities of *F. nucleatum* and *E. nodatum* between the two groups were not statistically significant (*p* > 0.05).

The green complex representative, *C. gingivalis*, was isolated in significantly higher quantities in children with generalized gingival inflammation (3.1 × 10^4^ ± 5.2 × 10^4^) (*p* < 0.05).

#### 4.2.2 Microbial associations

The results provide a rationale to suggest that specific combinations of microorganisms within subgingival microbial associations may also play a significant role in the clinical manifestation and severity of gingival tissue inflammation.

The following table presents data on the average number of species involved in subgingival microbial associations in children with localized and generalized gingival inflammation (Table 5).

**Table 5:** Average number of microorganisms in subgingival microbial associations.

МО BOP	Number of microorganisms involved in microbial associations	Independent T test
BOP up to 30%	2,25 ± 1,57	*t* *=* *−2,946 р* *<* *0,05*
BOP over 30%	4,07 ± 1,81	

The table shows that in children with localized gingival inflammation, microbial complexes typically consisted of an average of two microorganisms, whereas in those with generalized inflammation, the number of microorganisms involved was approximately twice as high—4.07 ± 1.81 (*p* < 0.05).

**Figure 1:**
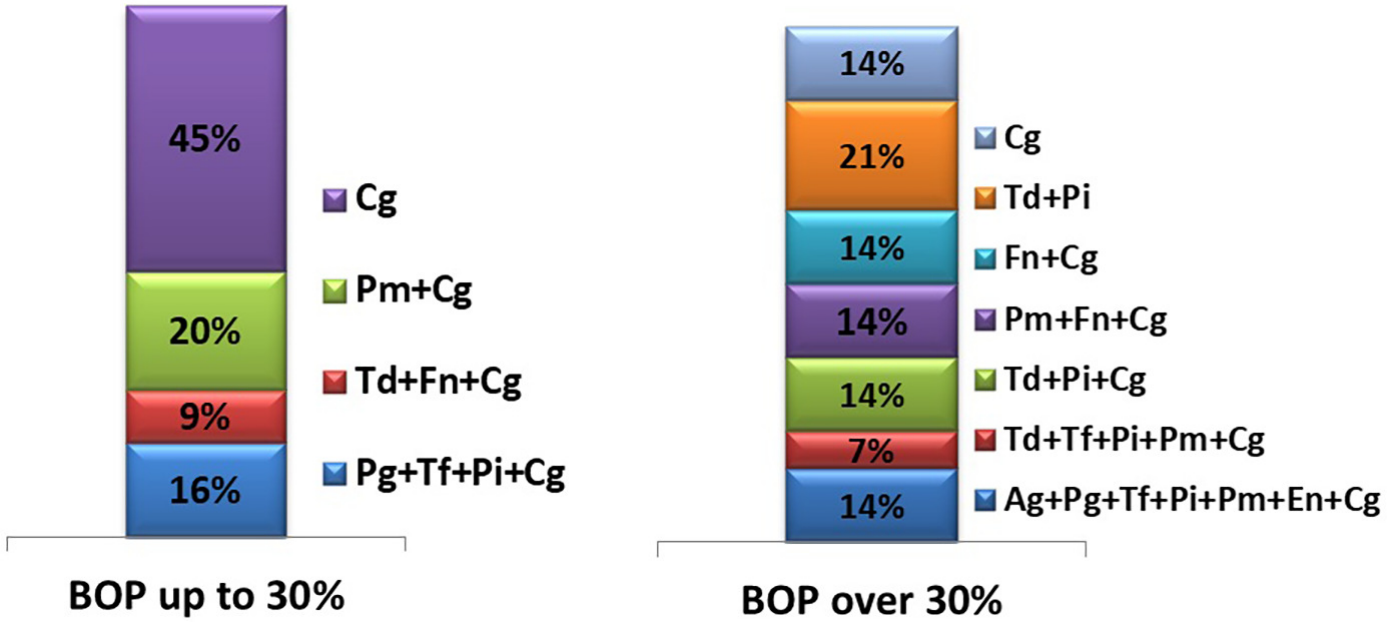
Types of microbial associations in children from both groups.

The following figure illustrates the types of microbial associations identified in children from both groups (Figure 1).

The figure clearly illustrates that microbial associations in children with localized gingival inflammation consist of fewer periodontal pathogens, in contrast to the more complex microbial associations observed in children with more severe inflammation.

In cases of localized gingival inflammation, the most frequently isolated pathogen was *C. gingivalis*, found alone in 45% of samples. Other combinations of microorganisms within microbial complexes ranged from 9% to 20%, with *C. gingivalis* participating in all of them.

In contrast, children with generalized gingival inflammation exhibited markedly more complex microbial associations, in some cases involving up to seven different species. Notably, red complex species (according to Socransky's classification) were always isolated in combination with orange complex microorganisms.

## 5 Discussion

The aim of the present study was to investigate the relationship between the severity of gingival inflammation and the subgingival microbial profile in children aged 12–14 years diagnosed with dental biofilm induced gingivitis.

The results revealed that children in both groups exhibited unsatisfactory oral hygiene habits, although children with localized gingival inflammation had a significantly lower number of tooth surfaces covered by plaque biofilm. Reiniger et al. state that regardless of the level of plaque accumulation, poorly established oral hygiene habits are a key factor in the progression of gingival inflammation to inflammatory-destructive involvement of periodontal structures (19).

Our study also found that the severity of gingival changes, whether localized or generalized, was comparable (SBI range: 1,02–1,37). This may be due to the methodology used, where sulcular bleeding was assessed orally in the first and third quadrants, regions where bleeding is generally minimal. This is due to the fact that, generally, less plaque is retained orally, leading to less inflammation. Additionally, the palatal gingiva is thicker, keratinized tissue which is why it is less likely to be affected by provoked bleeding.This likely led to an overall reduction in SBI scores. Similar patterns have been observed by other authors, although most studies in the literature focus on adult patients (20, 21).

We found that the total microbial load in children with generalized gingival inflammation was significantly higher than in those with localized inflammation. Given the poorer oral hygiene and gingival status in this group, this finding is not surprising and has also been reported by other research teams (22–24).

One notable observation in our study was that *A. actinomycetemcomitans* was not isolated in children with localized gingival inflammation. Papaioannou et al. conducted a study on 93 healthy children aged 3–12 and similarly reported no isolation of *A. actinomycetemcomitans* in any of the samples (25). This pathogen produces a range of virulence factors, the most notable being leukotoxin, which plays a central role in triggering pathological responses in gingival tissues. Literature suggests that in childhood and adolescence, the prevalence of this periodontal pathogen is minimal, particularly in cases of gingivitis or in healthy individuals. Some studies report isolation rates of 24% in children with gingivitis (26), while others show much lower prevalence rates (27–29).

Regarding the quantitative characteristics of isolated periodontal pathogens, we observed that *T. denticola* was isolated in significantly higher quantities in children with generalized gingival inflammation. Using advanced database analysis tools, Marotz et al. identified the *Treponema-to-Corynebacterium* ratio as a novel microbial indicator for periodontitis. Their study found this early indicator correlates not only with poor periodontal health, but also with cardiometabolic markers in the early stages of disease pathogenesis—a finding of particular relevance in childhood and adolescence (30). The results obtained by us indicate that more severe gingival inflammation in children is specifically associated with periodontal pathogens that are found in patients with inflammatory destructive changes in the periodontal space. This fact is of crucial importance for periodontal prevention in childhood.

The red complex, as described by Socransky, has been extensively studied due to its ability to impair host physiology through virulence factors that directly damage periodontal structures (31). Papaioannou et al. also found a significant effect of age on the proportions of red complex species. They proposed that the increase in *P. gingivalis* with age may be linked to higher levels of plaque and more severe gingival inflammation (25, 31). In our study, *P. gingivalis* and *T. forsythia* were detected in higher quantities in children with generalized gingival inflammation however, these differences were not statistically significant compared to the localized group.

Within the orange complex, we found that *P. intermedia* and *P. micros* were present in significantly higher quantities in cases of more severe inflammation (*p* < 0.05), whereas no significant differences were observed in the levels of *F. nucleatum* and *E. nodatum* (*p* > 0.05). These findings align with those of Albandar et al., who reported significantly higher isolation rates of *P. intermedia* in adolescents with active periodontal disease. Other authors have also linked aggressive periodontitis with elevated levels of *P. intermedia* and *P. gingivalis* (28, 32). Similarly, high levels of *P. intermedia* have been observed in adolescent girls with polycystic ovary syndrome and gingivitis (33).

Our study also found that a significantly greater number of microorganisms (on average ∼4) participated in microbial associations in children with generalized gingival inflammation. Furthermore, the composition of these associations was more complex and diverse than in children with localized inflammation. These patterns are consistent with findings from other studies, such as one involving patients who abstained from oral hygiene for 21 days, during which microbial complexity increased significantly (22).

Finally, it is important to note that in children with generalized gingival inflammation, red complex species were always isolated in combination with microorganisms from the orange complex, suggesting a synergistic relationship that may contribute to the pathogenesis of more severe forms of gingival disease.

## 6 Conclusion

This study demonstrated that as the severity of gingival inflammation increases, there is a corresponding increase in the quantity of subgingival periodontal pathogens. Moreover, the microbial diversity within the subgingival biofilm significantly increases with disease severity, providing further evidence for the critical role of microbial ecology in the pathogenesis of gingival inflammation in children.

The clinical significance of this study offering insights into the early detection and prevention of periodontal diseases in children by identifying the specific periodontal pathogens associated with severity of gingival inflammation. The obtained results point to the need for targeted prevention and early intervention strategies, which could significantly impact long-term oral health outcomes. Future research should continue to explore these findings, potentially leading to new diagnostic tools and therapeutic approaches in pediatric periodontal care.

## 7 Limitation

One limitation of this study is the small sample size, which may reduce the generalizability of the findings and limit the statistical power to detect smaller effects. A larger sample would provide more robust and reliable results, particularly in detecting subtle trends or patterns.

## 8 Future considerations

Future research should focus on diagnostic methods including more еasily applicable and accessible methods for general practicing dentists, refining personalized treatment strategies, and implementing preventive interventions at the earliest stages of gingival disease.

## Data Availability

The data that support the findings of this study contain personal information and are available from the corresponding author upon reasonable request. Access to the data will be granted in compliance with applicable data protection laws and regulations, ensuring the confidentiality and privacy of the individuals involved.
